# Feasibility Study on Temperature Distribution Measurement Method of Thrust Sliding Bearing Bush Based on FBG Quasi-Distributed Sensing

**DOI:** 10.3390/s19143245

**Published:** 2019-07-23

**Authors:** Hu Liu, Qiang Yu, Yuegang Tan, Wenjun Xu, Bing Huang, Zhichao Xie, Jian Mao

**Affiliations:** 1 School of Mechanical and Electronic Engineering, Wuhan University of Technology, Wuhan 430070, China; 2 China Ship Development and Design Center, Wuhan 430064, China; 3 School of Information Engineering, Wuhan University of Technology, Wuhan 430070, China

**Keywords:** thrust sliding bearing bush, FBG temperature sensor, measurement of temperature distribution

## Abstract

According to the characteristics of the temperature distribution of the thrust sliding bearing bush, the principle and method of quasi-distributed fiber Bragg grating (FBG) sensing is used to measure it. The key problems such as calibration, arrangement and lying of optical FBG sensors are studied by using the simulated thrust sliding bearing bush, which was customized in the laboratory. Combined with the thrust sliding bearing bush, the measurement experiments were carried out, which were divided into two groups: Steady-state experiments and transient experiment. The steady-state experiments obtain the temperature data measured by the FBG temperature sensors at each setting temperature, and the transient experiment obtains the relationship between the measured temperature by each temperature sensor and time in the heating and cooling process. The experimental results showed that the FBG temperature sensors had good accuracy, stability and consistency when measuring the temperature distribution of bearing bush.

## 1. Introduction

When the thrust sliding bearing is working, a hydrodynamic pressure oil film can be formed between the thrust-bearing bush and the thrust collar to bear the axial load of rotating machinery such as the ship propeller shaft, water turbine and generator set [1]. With the development of all kinds of machinery towards the direction of many parameters, high power and flexibility, and the increasingly complex working environment, the axial thrust required by thrust sliding bearing is constantly increasing. Therefore, it is of great significance to monitor the condition of thrust sliding bearing.

In the process of application, there are many serious accidents caused by the failure of thrust sliding bearing. For example, the Nanhai power plant in Japan in 1972, the Datong power plant in 1985, and the Fuxin power plant in 2000 and other accidents caused by different degrees of system failure of generator bearing rotors [2]. High temperature friction and deformation on the working surface of thrust sliding bearing bush caused bearing failure, such as the thrust sliding bearing of 3# and 4# circulating pump of 300 MW thermal power unit in Tianye power plant [3]. The thrust collar of a large extruder in a foreign country was broken due to high temperature [4,5]. Such cases are everywhere, it is mostly caused by the high temperature of thrust sliding bearing. In order to improve the service life of thrust sliding bearing and reduce the accidents caused by its high temperature failure, it is necessary to measure the bearing bush temperature of thrust sliding bearing on line.

At present, there are mainly the theoretical analysis, numerical simulation method and experimental research method about the temperature distribution measurement of thrust sliding bearing bush. The theoretical simulation method obtains the material properties and boundary conditions of the main components and related components of thrust sliding bearing, calculates the thermal conductivity, convection and radiation, and then obtains the temperature distribution data of thrust sliding bearing through simulation analysis [6,7,8]. However, the assembly relationship between key components of thrust sliding bearing and the complexity of lubricating oil flow field and thermal boundary conditions, the working environment of thrust sliding bearing cannot be truly reflected, and the accuracy of bearing temperature distribution data obtained through theoretical analysis is difficult to be guaranteed [9].

**Figure 1:**
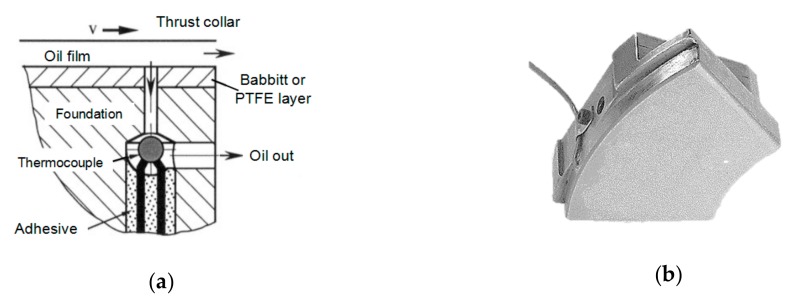
Installation of a thermocouple. (**a**) Installation schematic diagram; (**b**) installation physical drawing.

The experimental research method is mainly to measure the temperature of thrust sliding bearing through temperature sensors. In 2004, Glavatskih S.B. [10] studied a method for monitoring the temperature of the bearing oil film. The thermocouple was used to measure the temperature on the back of the bush liner and the bushing, and the thermocouple was installed as shown in Figure 1a,b. At the same time, this method was also used to measure the temperature of the thrust sliding bearing of the elastic metal plastic pad. In order to avoid damage to the surface of sliding friction pair of thrust sliding bearing, sensors are usually embedded and installed inside the thrust bearing. After A/D conversion, the measured temperature is displayed by relevant data acquisition and analysis software, so as to realize the purpose of real-time monitoring of thrust sliding bearing. However, the temperature distribution of thrust sliding bearing measured by traditional electrical sensors is not ideal due to electromagnetic interference, complex quasi-distributed measurement system and low measurement accuracy [11].

In addition, optical temperature measuring instruments such as infrared thermal imager are also used to monitor the temperature of sliding bearing. In 2011, Cristea A F et al. [12] proposed a thermocouple based method, which was proposed to determine the circumferential and axial temperature distributions of the lubricating film in the bearing groove under low load steady-state conditions through an experimental study. In 2016, Wei Wei et al. [13] carried out the sliding friction test of thrust joint bearing. When the bearing was subjected to transverse load, the surface temperature of the bearing and the working area temperature of the sliding friction pair were measured by infrared thermal imager and infrared thermometer. However, due to the high price, harsh measuring conditions and low measuring accuracy, it was rarely used in practice. As the existing measurement methods fail to meet the requirements of accurate measurement of the temperature distribution of thrust sliding bearing bush and optimization design of thrust sliding bearing material and structure, better measurement methods are still needed.

The unique anti-electromagnetic interference advantages of fiber Bragg gratings (FBGs) are incomparable to other electrical sensors. In addition, FBGs are small in size, good in stability, flexible in use and easy to integrate into a network, which makes them widely used in the field of temperature measurement [14,15,16]. In 2012, Hongjuan Xi [17] measured the temperature of transformer winding with FBG, indicating that the stress generated by oil flow inside the transformer will not affect the measurement of temperature by FBG. In 2013, Lei Tang [18] used FBG sensor to transform the temperature distribution stress of the rail into the change of the central wavelength of the FBG, realizing the online monitoring of the thermal expansion of the rail. In 2014, Jun Hong [19] measured the temperature distribution near the drill bit with FBG, and measured the temperature with multiple gratings, which truly reflected the real-time change of the temperature distribution near the workpiece in the drilling process. In 2015, Jiu Tan [20] used FBG sensor to realize real-time monitoring of temperature distribution in goaf of coal mine, and formed the production technology and method of the temperature measuring cable for coal mine.

In order to meet the demand of the sliding thrust bearing temperature distribution measurement, the sliding thrust bearing surface temperature measurement problem has been studied by using FBG temperature sensors, and the key problems such as the calibration, arrangement and laying of the optical fiber sensors have been solved, and a method for measuring the temperature distribution of thrust sliding bearing bush with quasi-distributed optical fiber sensing is presented. The effectiveness of this method is proved by experimental analysis.

## 2. Method of Measurement

### 2.1. Bearing Bush Structure and Working Charateristics

**Figure 2:**
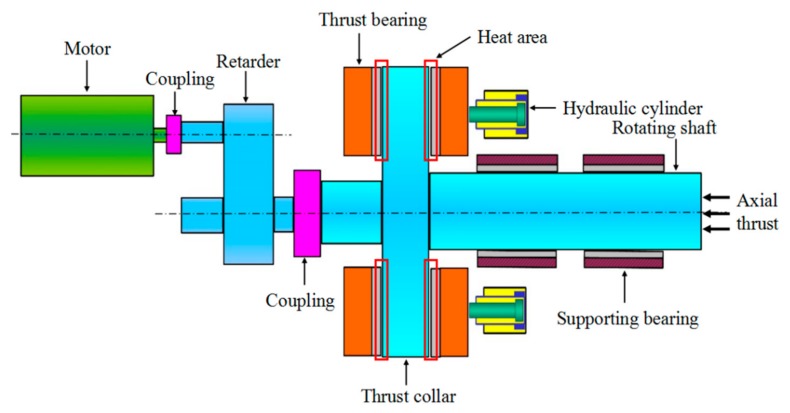
Structure diagram of thrust sliding bearing test bench.

The temperature distribution measurement method of thrust sliding bearing bush was designed to a certain type of thrust sliding bearing test bench. Figure 2 shows the test bench structure.

As shown in Figure 2, the thrust sliding bearing test bench is mainly composed of a motor, coupling, retarder, thrust collar, thrust bearing, hydraulic cylinder, rotating shaft and supporting bearing. The hydraulic cylinder is set at the right end of the rotating shaft, which can exert the axial thrust on the thrust sliding bearing.

The axial thrust loading device on the right end of the rotating shaft exerts axial thrust and transmits the axial thrust to the thrust collar through the rotating shaft. When the motor rotates and drives the thrust bearing through the reducer, the thrust bearing bush is stationary, and the thrust disk rotates with the rotating shaft. The force is transferred to the thrust bearing bush through the load of oil film between the thrust collar and the thrust bearing bush, and the thrust bearing bush bears the axial load of the whole unit.

**Figure 3:**
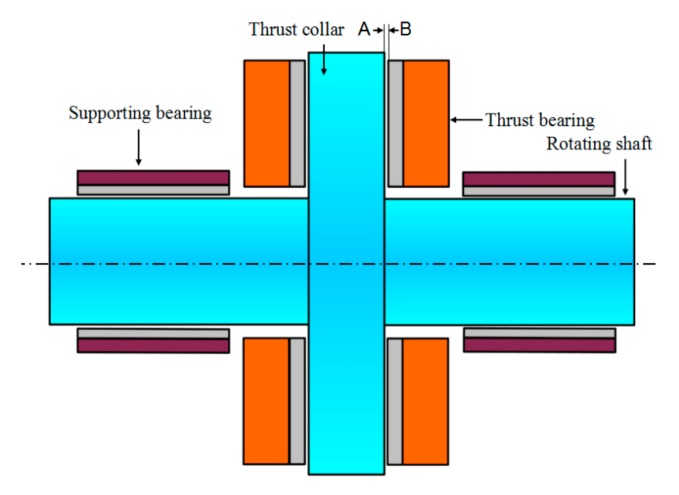
Internal structure of thrust sliding bearing.

In addition, the thrust bearing bush is one of the key internal structures of thrust sliding bearing, as shown in Figure 3. It is a schematic diagram of the internal structure of thrust sliding bearing. Plane A is the working face of the thrust collar, while plane B is the working face of the thrust bearing bush, and a sliding friction pair is formed between thrust collar face A and thrust bearing bush face B. When the thrust sliding bearing works, the axial thrust generated by the wedge-shaped oil film formed between the sliding friction pairs can be used to balance the axial external force of the thrust sliding bearing, and the wedge-shaped dynamic oil film will generate a lot of friction heat. Therefore, in the process of measuring the temperature distribution of bearing bush, the temperature distribution of the dynamic pressure oil film should be measured first. Obviously, traditional temperature sensors are difficult to measure directly.

### 2.2. FBG Quasi-Distribution Measurement Method for Temperature Distribution of Bearing Bush

Compared with traditional sensors, one of the characteristics of FBG sensors is that the quasi-distributed FBG sensors network can be composed by using multiplexing technology to measure multiple points in a wide range. This technology has been widely used in quasi-distributed measurement networks in various fields [21]. Optical multiplexing technology is based on the characteristics of light wave (subcarrier, delay time, encoding, reflection wavelength, etc.) and the transmission characteristics of fiber grating (high rate, low loss, etc.), while the transmission of optical signals in one or more optical fibers without interference. The appearance of FBG multiplexing technology, which greatly reduces the difficulty of setting up quasi-distributed measurement system, reduces the amount of wiring required in the past, reduces the volume of sensors and can be placed in a very small space for quasi-distributed measurement.

FBG wavelength division multiplexing (WDM) is the most common multiplexing technology. The principle of WDM is to connect several sensing gratings with different central wavelengths in series on a conducting fiber. In order to avoid interference between the measurement of each sensing grating, the drift range of the central reflection wavelength of each grating can be set by the offset of its central reflection wavelength.

**Figure 4:**
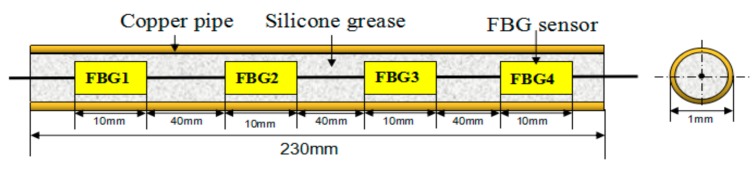
Schematic diagram of quasi-distributed fiber Bragg grating (FBG) temperature sensors.

Since the temperature distribution measurement of thrust sliding bearing bush requires many measuring points and has little available space, the FBG quasi-distributed measurement method is of great significance in the temperature distribution measurement of thrust sliding bearing bush. Figure 4 shows the schematic diagram of quasi-distributed FBG temperature sensors designed, According to Shihai Liu’s [22] analysis of oil film temperature distribution of thrust bearing shell by algorithmic processor description language (APDL) technology and the principle of economic applicability, the measurement method designed in this paper consists of three FBG temperature sensors, each of which has four temperature measuring points.

### 2.3. FBG Temperature Sensors Laying

The thrust bearing bush was composed of a steel foundation, metal wire pad and modified poly tetra fluoro ethylene (PTFE) plate. From the production process of thrust bearing bush, it could be known that the thrust bearing bush was made of an elastic metal composite layer and steel tile blank through tin brazing.

**Figure 5:**
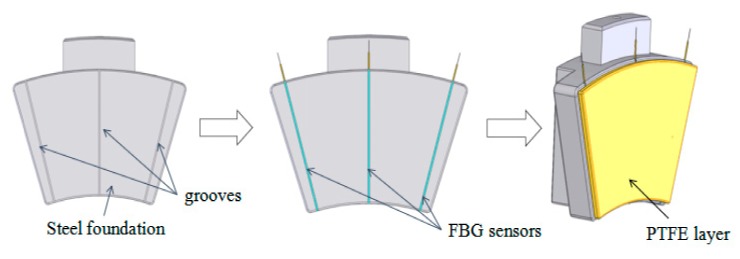
Installation process flow of FBG temperature sensors for thrust sliding bearing bush.

Figure 5 shows the installation process flow of FBG temperature sensors for the thrust sliding bearing bush. Due to the thrust sliding bearing was composed of an elastic metal composite layer and steel embryo by tin soldering hot-pressing, in front of the tin soldering process, three grooves with a section size of 3 mm × 3 mm were radially opened on the steel foundation fan-shaped surface, and would have good encapsulated with capillary tubes quasi-distributed FBG temperature sensors installation fixed in the grooves, at last, through the elasticity compound layer and metal tin soldering process steel tile embryo hot pressed into. Since the FBG is easily damaged in the dynamic environment inside the bearing, the packaging technology can better protect the FBG. As shown in Figure 4, the quasi-distributed fiber grating temperature sensor is fixed at one end and freely telescopic at the other end. It is encapsulated in a thin metal copper tube by heat-conducting silicon grease. The free expansion of one end ensured that the accuracy of the measuring temperature of fiber Bragg grating would not be disturbed by deformation and other factors during heating.

## 3. Measurements and Experimental Research

### 3.1. Preparation of FBG Measuring Body for Thrust Sliding Bearing Bush

**Figure 6:**
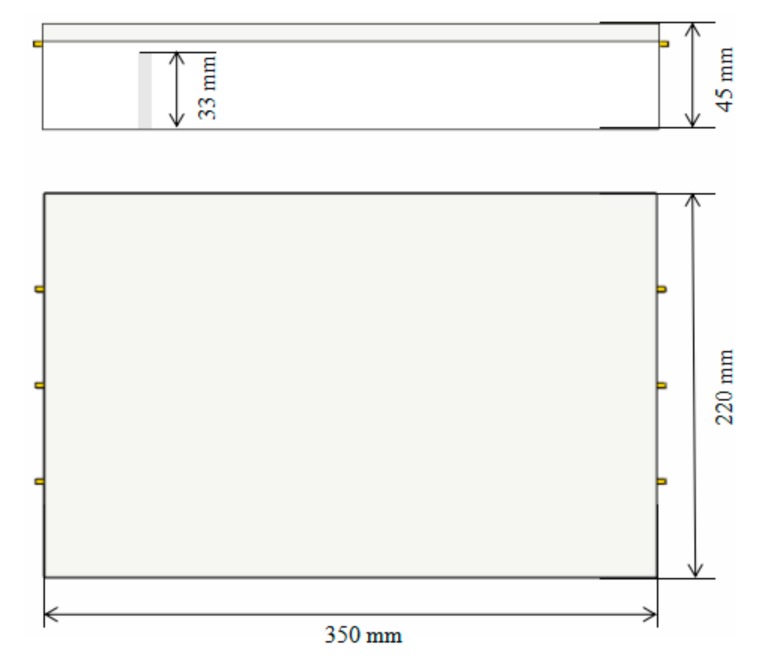
Dimension diagram of imitation thrust bearing bush.

**Figure 7:**
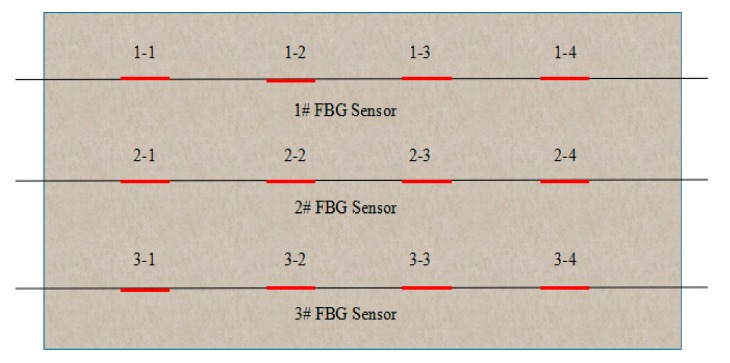
Schematic diagram of the measurement point of FBG temperature sensors.

As can be seen from the above, the FBG temperature sensors designed in this paper were arranged on the interlayer of steel tile billet and elastic metal composite material. Since this experiment could not be carried out directly on the prototype, temperature distribution and the purpose of this study was to verify the feasibility of fiber grating temperature sensor to measure the temperature distribution of thrust sliding bearing bush, other external dimensions have little influence on the measurement results when the elastic metal and plastic layer was consistent with the thickness of the prototype. Therefore, an imitation thrust sliding bearing bush (length, width and height: 350 mm × 220 mm × 45 mm) was prepared in this experiment. In the process, a blind hole (diameter: 2 mm, depth: 33 mm) was drilled on the steel base at the back of the measuring point 1-1, and a thermocouple was embedded in the experiment for comparison, as shown in Figure 6. The measuring points distribution of FBG temperature sensors used in this paper is shown in Figure 7.

### 3.2. Calibration of FBG Temperature Sensors

**Figure 8:**
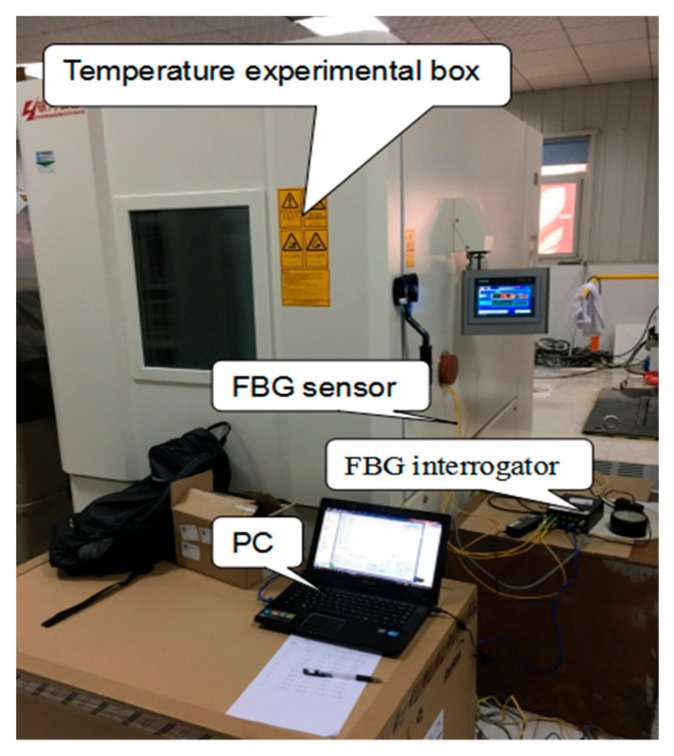
Calibration experiment system of the FBG temperature sensors.

The calibration experimental system of FBG sensors, as shown in Figure 8, was built. The experimental system was composed of a thrust bearing bush, interrogator, temperature experimental box, personal computer (PC) and FBG temperature sensors. The temperature uniformity of the temperature test box was ±2 °C, and the temperature fluctuation was ±0.5 °C, the resolution of the FBG interrogator was 1 pm. The thrust bush encapsulated with FBG temperature sensors was placed in the temperature experimental box to control the temperature. The central wavelength of the grating reflection was collected and recorded by the FBG interrogator and computer.

The temperature experimental box was opened at room temperature, and the first setting temperature was 50 °C, and the last setting temperature was 120 °C, the heating distant temperature was 10 °C. The temperature of the temperature experimental box needed 1.5 h to stabilize at each setting temperature point. When the data of the temperature experimental box were stabilized in a certain temperature point, the central wavelength data reflected by each fiber grating were collected and recorded, and the real-time temperature data were recorded at the same time. The sensitivity formula of FBG temperature sensors can be expressed as:

(1) $$S=\frac{\Delta \lambda }{\Delta T},$$

where *S* is the sensitivity of the FBG temperature sensors, Δλ is the wavelength change and ΔT is the temperature change. The sensitivity of each FBG temperature sensors measuring points can be obtained according to Equation (1), and the sensitivity of each measuring point is shown in Table 1. Among them, the first FBG temperature sensor sensitivity slants big reason is that the first FBG temperature sensor encapsulation mode is different from the other two, the first sensor is used for measuring the pressure (enterprise need to measure pressure and temperature at the same time), during measuring the temperature distribution, it was used to measure the temperature.

### 3.3. Experimental Test

**Figure 9:**
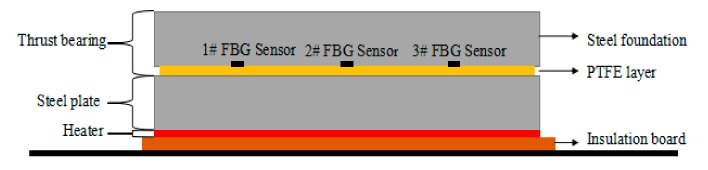
Experimental heating schematic diagram of thrust sliding bearing bush temperature distribution measurement.

**Figure 10:**
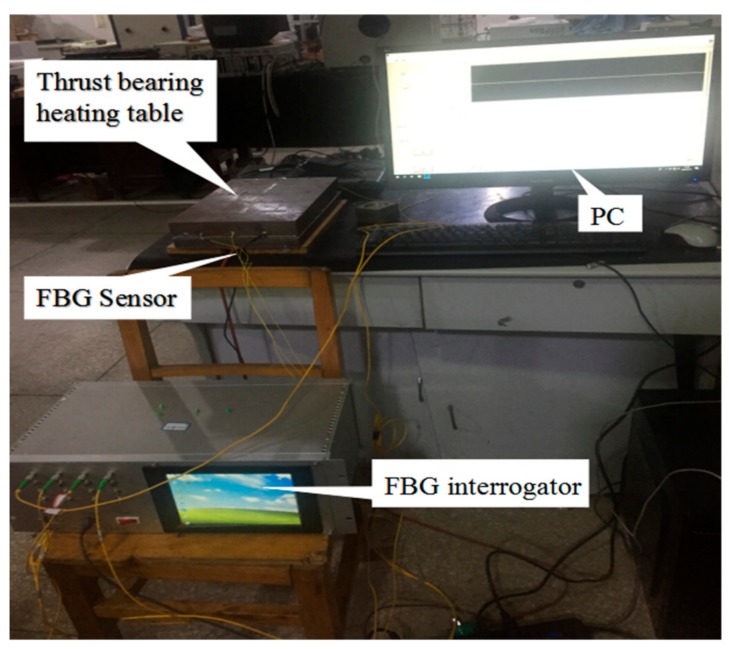
The architecture of the testbed.

In order to simulate the heating environment of the thrust sliding bearing bush, the schematic diagram of the experimental platform designed in this paper is shown in Figure 9. The experimental platform is composed of the insulation board, heater, steel plate and the bearing bush of the thrust sliding bearing, and the combination of heating plate and steel plate can simulate the heat of the oil film on the prototype machine. The steel plate acted as a temperature buffer according to the measurement point of fiber Bragg grating sensor, the results showed that the temperature was dynamically stable and the temperature fluctuation was small. If without the steel plate, the temperature fluctuation would be large, which is not conducive to the experiment. The surface size of heating element and the steel plate was consistent with the surface size of the bearing bush of thrust imitation. Figure 10 shows the spot figure of thrust sliding bearing bush temperature distribution measurement.

This experiment was conducted in a closed room with small air flow, and the room temperature was 20 °C (±0.5 °C), The thrust bearing temperature was within 0–120 °C, and the measurement accuracy was required to be less than 2 °C. In the process of experimental measurement, the setting temperature of the heater was set to the a target value, and the wavelength of the FBG temperature sensors laid between the foundation and PTFE layer was measured by the FBG interrogator. The sampling frequency was 1 Hz. The initial wavelength of the FBG temperature sensors used in this paper is shown in Table 2.

### 3.4. Experimental Results and Analysis

**Figure 11:**
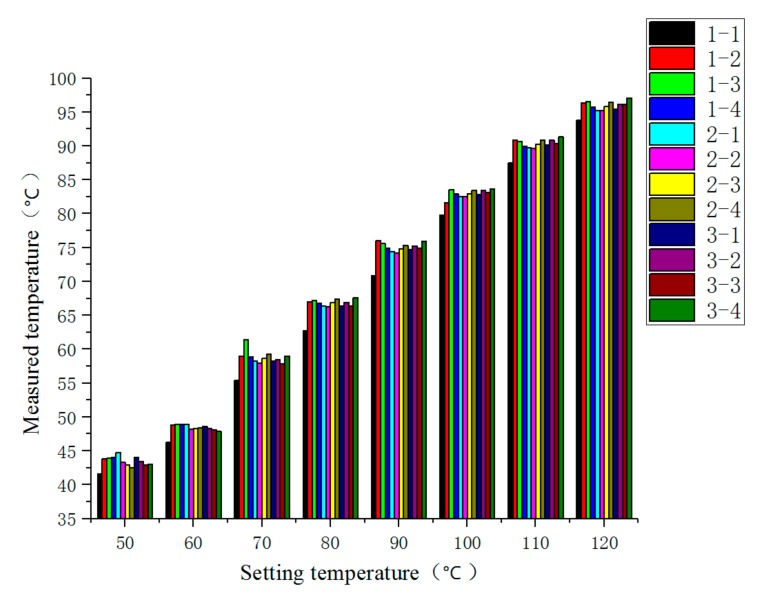
Steady-state measurement results of the temperature at each measuring point.

**Figure 12:**
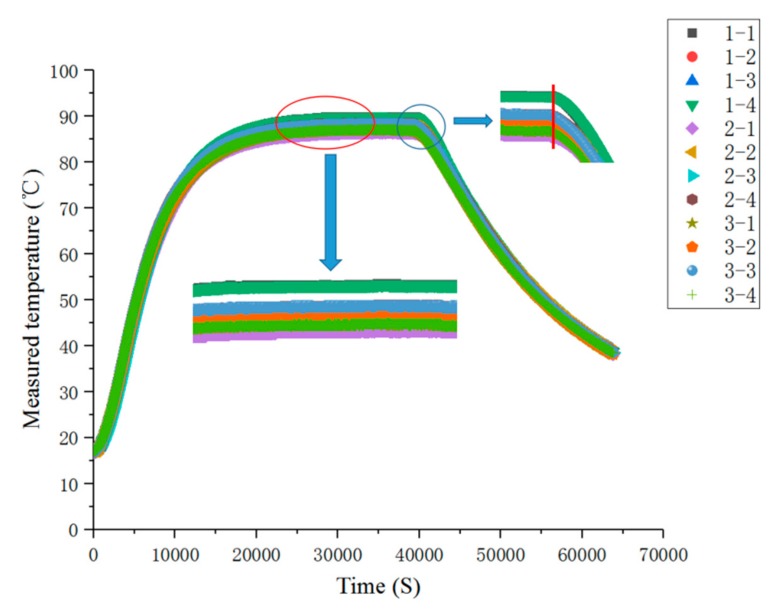
Transient measurement results of the temperature at each measuring point.

**Figure 13:**
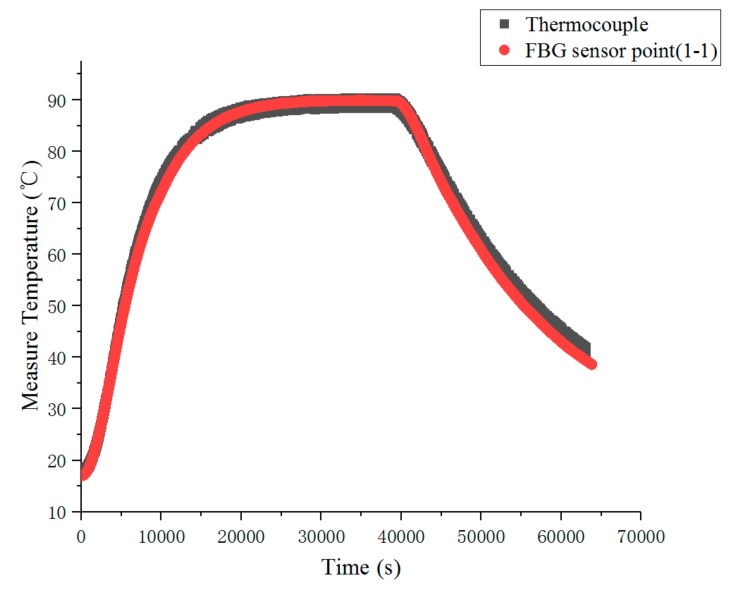
Comparison of measurement results of the thermocouple and measuring point 1-1 of FBG sensor.

The experiment was divided into steady-state temperature distribution experiments and transient temperature distribution experiment. Before the experiment, the preparation for the experiments was done, and the FBG temperature sensor, FBG interrogator, PC and heater power supply were connected in turn. When steady-state temperature distribution experiments were carried out, the heater temperature of the heater was set to 50, 60, 70, 80, 90, 100, 110 and 120 °C in turn at each experiment, and the temperature distribution was stable after 10.5 h of heating. When transient temperature distribution experiment was carried out, the temperature of the heater was set to 110 °C during the experiment. After heating for 10.5 h, the heater power supply was disconnected to stop heating. The wavelength of each FBG temperature sensor was recorded, and its temperature value was calculated. The steady-state temperature measurement results of each FBG temperature sensor are shown in Figure 11. The steady-state results of temperature measurements are shown in Figure 12, the comparison results of thermocouple and measuring point 1-1 of FBG sensor are shown in Figure 13.

As can be seen from Figure 11, at different setting temperatures, the measured temperatures at the same measuring points of the FBG temperature sensors were positively correlated with the setting temperatures, and due to the presence of steel plate, the measured temperatures at each measuring point were smaller than the set values. In addition, at the same setting temperatures, there was a small difference in the measurement results of the FBG temperature sensor, which was caused by the different heat dissipation conditions of each measurement point.

As can be seen from Figure 12, when the heating time reached about 20,000 s, the temperature distribution reached the steady state, and the consistency of each sensor measurement point was better. When the heating stopped at about 40,000 s, the response of each sensor was faster and the consistency was better when the temperature dropped.

As can be seen from Figure 13, compared with the thermocouple, the FBG sensor had no lag, and the measurement results were consistent with the thermocouple measurement results. The measurement curve of the FBG sensor was thinner than that of thermocouple, which indicates that the measurement of the fiber optic sensor was more stable than that of the thermocouple.

**Figure 14:**
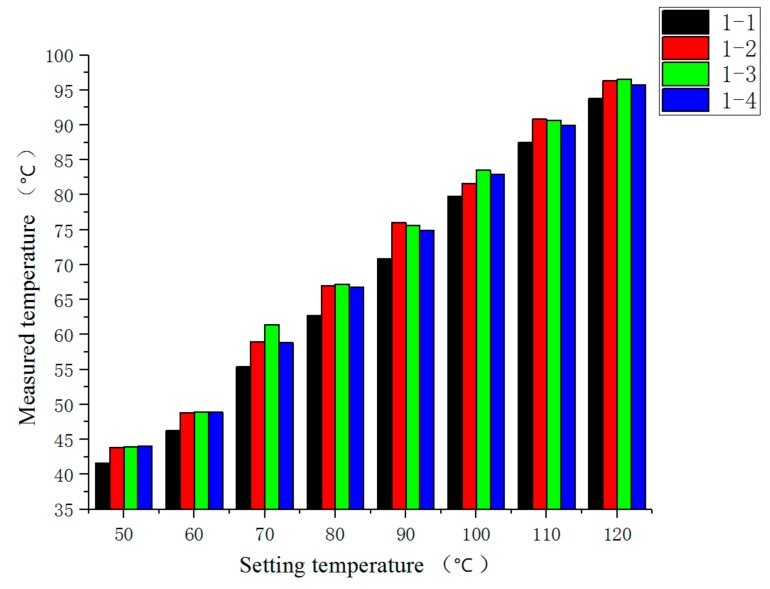
Comparison of the steady-state temperature measured at four measuring points of 1# FBG temperature sensor.

**Figure 15:**
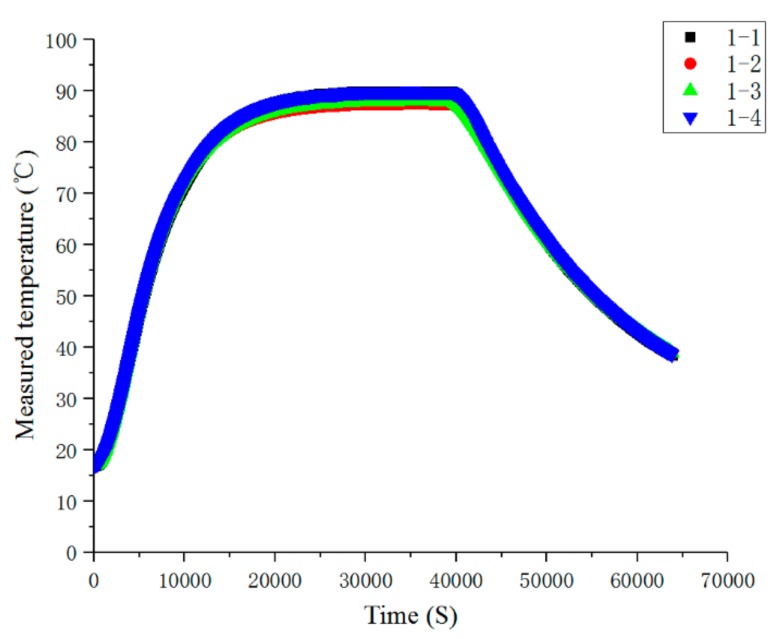
Comparison of the transient temperature measured at four measuring points of 1# FBG temperature sensor.

In the steady-state temperature distribution experiment, the steady-state temperature measured at four measuring points of 1# FBG temperature sensor is shown in Figure 14. In the experiment of transient temperature distribution, the transient results of the temperature measured at four measuring points of 1# FBG temperature sensor are shown in Figure 15.

It can be seen from Figure 14 that the measured temperature at the measuring point 1-1 was about 2 °C lower than that at the other three measuring points, or so, and the 1-2, 1-3 and 1-4 three measuring points measured temperature gap was smaller, The possible reason is that the measuring point 1-1 was closer to the aisle, and the heat dissipation condition was better than the other three measuring points, while the heat dissipation condition of the other three measuring points was roughly the same.

It can be seen from Figure 15 that the trend of temperatures measured by the four sensor points were consonant with each other during the heating and cooling stages. In the stage of temperature stabilization, there was a small gap between the temperatures measured by the four sensors, which was caused by different heat dissipation conditions.

**Figure 16:**
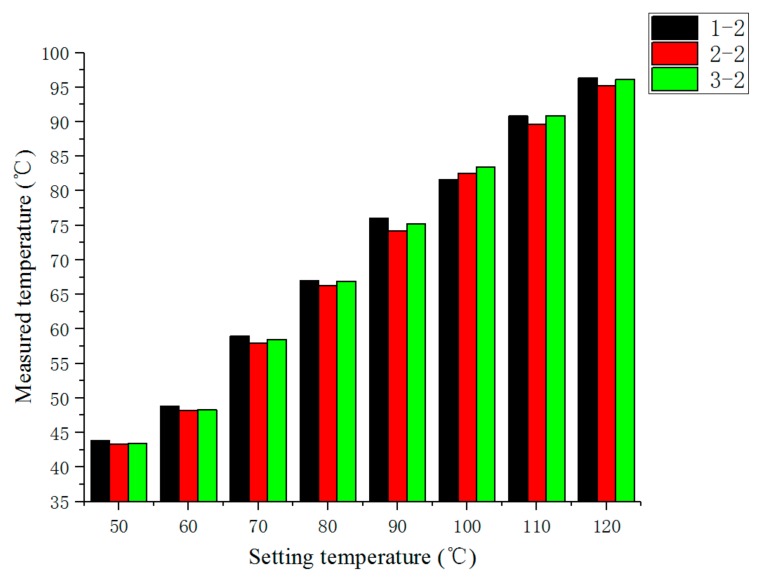
Comparison of the steady-state temperature measurements at the second measuring point of each temperature sensor.

**Figure 17:**
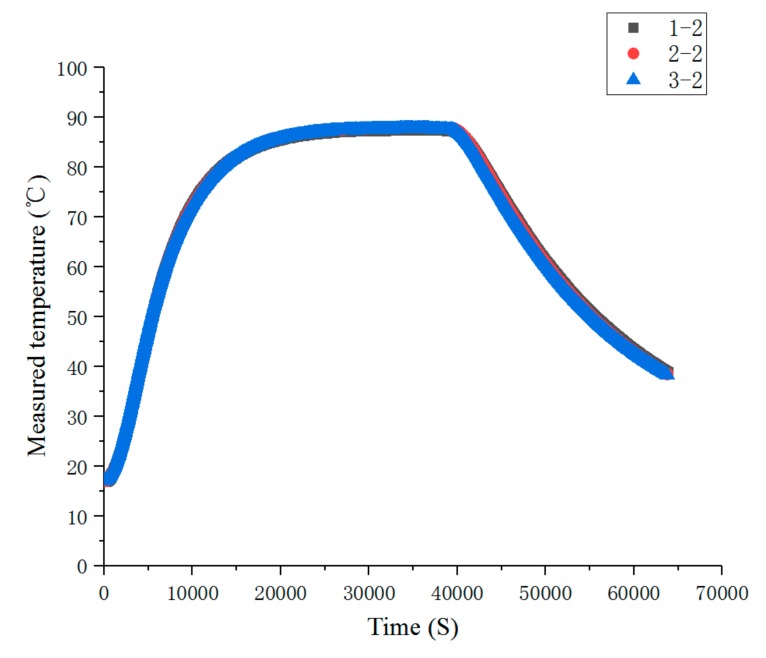
Comparison of the transient temperature measurements at the second measuring point of each temperature sensor.

Figure 16 is a steady-state comparison of temperature measured at the second measuring point of each temperature sensor. It can be seen from Figure 16 that when the heat dissipation conditions were roughly the same, the difference of measured temperature at each measuring point was relatively small. Figure 17 is a transient comparison of temperature measured at the second measuring point of each temperature sensor. From Figure 17, we can still see that when the heat dissipation conditions were roughly the same, the temperature difference between the measured points was small.

## 4. Results

In this paper, the temperature distribution measurement method of the thrust sliding bearing bush based on quasi-distributed FBG sensing technology was studied. Finally, based on the temperature measurement method and the custom thrust imitation sliding bearing bush, the temperature measurement tests were carried out.

The experimental process and results showed that FBG temperature sensors had good accuracy, stability and consistency in measuring the temperature distribution of sliding bearing bush, its temperature response was rapid, and compared with the results of thermocouple measurement, the measurement error of FBG temperature sensor was less than 2 °C. Therefore, the temperature distribution measurement method based on FBG temperature sensors could effectively complete the measurement of the temperature distribution of the thrust sliding bearing bush within a certain accuracy. This method could provide a basis and technical support for the subsequent measurement of the temperature distribution of the thrust sliding bearing bush.

## Figures and Tables

**Table 1:** Calibration results of measurement points of each FBG temperature sensors.

Number of Measuring Points	The Temperature Sensitivity (pm/°C)	Linearly Correlation Coefficient
1-1	27.520	0.9993
1-2	24.846	0.9999
1-3	24.945	0.9998
1-4	25.129	0.9996
2-1	10.477	0.9991
2-2	10.557	0.9992
2-3	10.589	0.9989
2-4	10.614	0.9991
3-1	10.556	0.9992
3-2	10.431	0.9991
3-3	10.552	0.9991
3-4	10.369	0.9990

**Table 2:** Initial wavelength of each measurement point of the FBG temperature sensors.

Number of Sensors	Point 1	Point 2	Point 3	Point 4
1# sensor	1535.873	1540.021	1543.967	1547.859
2# sensor	1535.955	1539.970	1543.945	1547.940
3# sensor	1535.991	1539.962	1543.959	1547.913
